# Subarachnoid hemorrhage and cardiac arrest: should every resuscitated patient receive cranial imaging?

**DOI:** 10.1186/cc9718

**Published:** 2011-03-11

**Authors:** C Leithner, D Hasper, CJ Ploner, C Storm

**Affiliations:** 1 Charite Universitaetsmedizin, Berlin, Germany

## Introduction

Intracranial hemorrhage, especially subarachnoid hemorrhage (SAH), may lead to cardiac arrest via a number of mechanisms. A recent prospective Japanese study found 16.2% of patients with SAH among those resuscitated from out-of-hospital cardiac arrest (OHCA) [1]. In contrast, a retrospective European study found only 4% and the majority of patients had symptoms suggestive of SAH prior to OHCA [2]. Hence, different recommendations regarding routine cranial imaging may be obtained from the two studies.

## Methods

We therefore evaluated retrospectively the rate of SAH in cardiac arrest patients consecutively admitted to our internal medicine ICU. For all patients, CCT and autopsy findings were obtained, if available. In addition we screened emergency room or final medical reports of SAH patients admitted to our neurosurgical ICU for OHCA and resuscitation.

## Results

Cranial computed tomography (CCT) was performed in 129 of 421 (32.6%) cardiac arrest patients admitted to our internal medicine ICU, commonly on the day of admission (52% of CCTs) or within the first week (85%). None of the CCTs showed signs of SAH. Retrospective analysis of all autopsies (*n *= 18) revealed no postmortem diagnosis of SAH. A retrospective analysis of SAH patients admitted to our neurosurgical ICU revealed only one out-of-hospital resuscitation among 141 SAH patients (0.7%), in line with a recent study [3].

## Conclusions

Our data indicate a low rate of SAH in patients with OHCA, especially when not clinically suspected. For our patient cohort, routine CCT may not be indicated after cardiac arrest. The rate of SAH leading to OHCA seems to differ significantly between Japan and Germany. Our results have to be interpreted with care because of the retrospective study design and possible selection bias. Further prospective studies are needed to confirm the results.
